# A Two-Stage Deep Learning Method for Non-Invasive Sow Body Temperature Prediction Fusing Thermal Imaging and Environmental Parameters

**DOI:** 10.3390/ani16111692

**Published:** 2026-05-31

**Authors:** Shengyong Xu, Ziyi Qin, Qiao Huang, Chen Tan, Xuewen Xu, Xuan Li

**Affiliations:** 1 College of Engineering, Huazhong Agricultural University, Wuhan 430070, China; xsy@mail.hzau.edu.cn (S.X.); 2776608351@webmail.hzau.edu.cn (Z.Q.); huangqiao0628@163.com (Q.H.); 2 Ministry of Agriculture Key Laboratory of Agricultural Machinery for the Middle and Lower Reaches of the Yangtze River, Wuhan 430070, China; 3 The College of Animal Science & Technology and College of Veterinary Medicine, Huazhong Agricultural University, Wuhan 430070, China; tanchen@mail.hzau.edu.cn (C.T.); xuewen_xu@mail.hzau.edu.cn (X.X.)

**Keywords:** sow body temperature, non-invasive temperature measurement, infrared thermal imaging, multimodal data fusion, deep learning, computer vision, precision livestock farming, rectal temperature prediction, YOLO, random forest

## Abstract

Traditional rectal temperature measurement in pigs induces stress in animals, imposes a heavy labor burden on staff, and increases the risk of cross-infection. This study proposes a non-invasive deep learning approach to predict porcine rectal temperature by combining infrared thermal images of thermal windows with environmental parameters. A multimodal dataset is constructed by synchronously collecting thermal images, environmental parameters, and actual rectal temperatures. Mask Region-based Convolutional Neural Network (Mask R-CNN), You Only Look Once version 8 small (YOLOv8s), and YOLOv11s are employed to automatically detect or segment thermal window regions, from which the maximum temperature of each region is extracted. To enhance model generalization under varying environmental conditions, a two-stage hybrid regression framework is established. In this framework, a Convolutional Neural Network (CNN) extracts spatial features from thermal images, a fully connected network (FCNN) encodes regional surface temperatures and environmental parameters, and a Transformer module captures cross-modal dependencies to generate a preliminary prediction. Subsequently, a Random Forest (RF) regressor is applied for residual correction and final output optimization. Comparative experiments on single-region, dual-region, and triple-region combinations demonstrate that the “eye + vulva” dual-region scheme yields the optimal performance, with a mean absolute error (MAE) of 0.1796 °C and a coefficient of determination (R^2^) of 0.8212. The prediction error of this scheme is reduced by 42.3% compared with the best-performing unimodal model. The proposed method provides a fast, accurate, and stress-free solution for porcine body temperature monitoring, thereby supporting the development of intelligent health management in livestock farming.

## Simple Summary

Traditional rectal temperature measurement in pigs is stressful, time-consuming, and carries a risk of cross-infection. This study proposes a non-invasive method based on infrared thermography to acquire thermal images of the pig’s ear, eye, and vulva regions, while ambient temperature, humidity, wind speed, and illumination are recorded simultaneously. An image processing algorithm is employed to automatically identify the target regions and extract their maximum temperature values. A two-stage artificial intelligence model is established to integrate thermal distribution features with environmental parameters for predicting rectal temperature. Experimental results demonstrate that the combination of the eye and vulva achieves optimal prediction accuracy, with an average error of 0.18 °C. The proposed method enables rapid and stress-free body temperature monitoring in pigs, thereby promoting the development of automated and animal-friendly livestock health management systems.

## 1. Introduction

Body temperature is a critical indicator of health status in pigs [1,2]. According to data from the National Bureau of Statistics of China, the national hog inventory reached 434 million head at the end of 2023, with the large-scale breeding rate of hogs exceeding 68%. Large-scale hog farms (≥500 head) have become the mainstay of hog production. Approximately 80% of pig diseases are associated with fever, and abnormal body temperature often precedes other clinical signs, particularly during the early stages of infectious diseases [3]. As the industry-standard approach, rectal temperature measurement involves complex procedures, is labor-intensive and time-consuming, and may induce stress responses in pigs while increasing the risk of cross-infection, rendering it unsuitable for real-time, high-frequency monitoring in large-scale pig farms [4]. Achieving accurate non-contact estimation of rectal (core) temperature in livestock thus remains a critical technical challenge in modern large-scale farming systems.

Infrared thermal imaging generates temperature distribution maps by capturing thermal radiation signals from the target surface. It offers distinct advantages such as being non-invasive, low-stress, and capable of real-time monitoring, thereby providing an effective alternative to the shortcomings of conventional thermometry [5,6]. The body surface of pigs contains “thermal window” regions that are highly correlated with core body temperature, making them ideal targets for infrared temperature measurement. Specific sites, such as the ear base, the ocular region, and the vulva, exhibit strong correlations with rectal temperature and are thus optimal locations for constructing non-contact body temperature monitoring systems [7,8]. However, thermal readings from different anatomical regions show differential sensitivity to environmental factors, which is the primary source of measurement bias. Wind speed substantially lowers ear temperature via convective heat loss from this exposed, thinly haired area, whereas the vulva, shielded by skin folds and a dense vascular network, is minimally affected [9]. High humidity suppresses evaporative cooling from the moist eye and vulva, elevating their surface readings, while the relatively dry ear surface remains largely unaffected. Ambient temperature influences all regions but with heterogeneous magnitude: the ocular region, rich in superficial vessels and low in thermal inertia, responds fastest and most strongly; the ear shows an intermediate response; and the vulva, buffered by subcutaneous fat, provides the most stable readings [10]. Currently, infrared thermal imaging has been applied in several aspects of pig production, including estrus detection, monitoring of piglet hypothermia and the risk of crushing, and the assessment of disease and heat stress, serving as an important sensing tool for precision pig farming [11,12]. However, infrared thermal imaging captures only the surface thermal radiation of pigs, which is highly susceptible to the above-mentioned environmental factors, leading to significant deviations from core rectal temperature. Consequently, it cannot directly replace rectal temperature to reflect the true health status of the animals [13,14]. Accurate retrieval of core body temperature must therefore rely on algorithmic modeling. To overcome these limitations, both machine learning and deep learning approaches have been employed to indirectly estimate core body temperature through computational models.

Machine learning algorithms, owing to their advantages in small-sample adaptability and interpretability, have been widely applied in early studies for indirect estimation of core body temperature. For example, Shen et al. extracted the maximum temperatures from the head and leg regions of broilers using infrared thermal imaging and combined them with environmental parameters, including ambient temperature, relative humidity, and light intensity, to construct a multiple linear regression model, achieving an average relative error of 0.33% [15]. Li et al. developed a comparative framework incorporating elastic net, random forest, XGBoost, and LightGBM models for dairy cow temperature prediction, in which a GWO-XGBoost model based on trunk infrared temperature and production performance data achieved an MAE of 0.232 °C and an RMSE of 0.294 °C [16]. Xiong et al. employed a Lasso regression model to predict rectal temperature in neonatal piglets, reducing the standard error of prediction (SEP) to 1.5 °C [17]. Tian et al. applied multiple linear regression to fit pig body temperature under varying environmental conditions, with a maximum error of 3.06% and an average absolute error of 1.41% [18]. In the medical field, Liang et al. constructed a nomogram-based risk prediction model for intraoperative hypothermia, demonstrating good discriminative ability and calibration performance [19]. These methods feature simple structures and high interpretability, but suffer from three core limitations: First, most studies generally have small sample sizes (mostly <500 samples) and fail to cover complex scenarios in real commercial farms such as seasonal fluctuations and breed differences, resulting in insufficient generalization ability. Second, they only rely on manually extracted numerical features such as the maximum temperature of specific regions, without utilizing the spatial thermal distribution information of infrared images, thus failing to capture subtle physiological temperature changes. Third, traditional linear and tree-based models have limited ability to model the complex nonlinear relationships among body surface temperature, environmental interference and core body temperature, leading to a significant decline in accuracy under dynamic farming conditions.

With the rapid advancement of deep learning, its powerful end-to-end feature learning capability has significantly improved the accuracy and generalization of livestock body temperature prediction models. Gorczyca et al. compared deep neural networks, gradient boosting trees, and random forests for piglet temperature prediction, showing that deep neural networks achieved a relative error of only 0.36%, significantly outperforming generalized linear regression models [20]. Yan et al. employed a Res2Net model to estimate the core body temperature of laying hens, achieving an R^2^ of 0.956 on the test set [21]. Shu et al. utilized random forests, gradient boosting machines, and artificial neural networks to predict dairy cow temperature, where the artificial neural network achieved an RMSE of 0.3 °C for vaginal temperature prediction, outperforming regularized linear regression [22]. Basak et al. reported that a multilayer feedforward neural network trained with the Levenberg–Marquardt algorithm outperformed multiple linear regression in modeling pig surface temperature, with the temperature–humidity index identified as a key input variable [23]. He et al. developed a skeleton-based tree model for automatic detection of ocular temperature in dairy cows using infrared thermal imaging, achieving an MAE of 0.35 °C on the test set [24]. Wang et al. further demonstrated that maximum temperatures of the eye and vulvar regions can effectively characterize estrus-related temperature variations, achieving recognition rates of over 80% using Logistic regression and SVM models [25].

In summary, compared with traditional linear and shallow models, deep learning approaches exhibit superior capability in capturing complex nonlinear relationships, leading to improved prediction accuracy and robustness. However, current studies on non-invasive body temperature prediction in pigs still face notable limitations. From a technical perspective, most existing methods rely on a single modality and fail to effectively integrate infrared image features with environmental parameters, resulting in limited prediction performance and weak generalization under complex farming conditions. From a scientific perspective, current research predominantly focuses on single thermal windows, lacking systematic investigation of multi-region combinations; hence, the optimal measurement strategy remains unclear.

Unlike previous studies that relied on the maximum temperature of a single thermal imaging window, employed shallow regression models, and failed to systematically integrate environmental parameters or optimize multi-region combinations, this study focuses on the optimization of multi-source data fusion and temperature measurement strategies, and proposes a non-invasive body temperature prediction framework for sows in complex farming environments [26,27]. The main contributions of this study are summarized as follows:

(1) A multimodal data fusion framework is developed to integrate spatial features from infrared images, maximum temperatures from key body surface regions, and environmental parameters, enabling a unified representation of heterogeneous information and improving robustness under environmental disturbances.
(2) A two-stage prediction strategy is proposed, in which an initial prediction is generated using deep learning models and subsequently refined through ensemble learning, effectively reducing model variance and improving prediction stability and generalization performance in complex environments.
(3) A systematic analysis of thermal window combinations demonstrates that the “eye + vulva” dual-region configuration outperforms three-region combinations, suggesting that redundant thermal information can negatively affect prediction accuracy and providing guidance for optimal selection of temperature measurement sites.

## 2. Materials and Methods

### 2.1. Experimental Subjects and Equipment

To systematically investigate the effects of surface thermal characteristics and environmental parameters on the core body temperature of sows, two rounds of data collection were conducted in breeding pens located at the Zhuletianyuan No. 1 and No. 2 production zones, Xinzhou District, Wuhan, Hubei Province, China. The first round took place from 15 May to 10 June 2024, and the second from 11 January to 25 January 2026. A dataset with multi-temporal and multi-environmental variability was thus established.

The pig house adopted a sloped-roof confined housing system, equipped with an efficient ventilation system and a combination of natural and artificial lighting. The experiment adopted a stratified random sampling design. The experimental subjects were uniform second-parity, non-pregnant multiparous Landrace sows with an average body weight of 250 kg. Sampling was conducted via stratified random selection based on time periods and pen locations to eliminate the confounding effects of parity, reproductive status, and pen microenvironment.

The infrared thermal imaging device used in this study was the Guide IR IPM630 (Wuhan Guide Sensmart Tech Co., Ltd., Wuhan, China). Equipped with an uncooled focal plane detector, it offers high stability and adaptability to livestock production environments. Its main technical specifications are as follows: infrared resolution of 640 × 512 pixels, noise equivalent temperature difference (NETD) ≤ 40 mK at 30 °C, temperature measurement range of 20–50 °C, and effective shooting distance of 0.5–3.5 m. The device supports software integration, facilitating system configuration and secondary development.

A portable environmental parameters acquisition device was custom-built to monitor ambient temperature, humidity, wind speed, and light intensity inside the pig house. The system integrates high-precision temperature and humidity sensors, an ultrasonic wind speed sensor, and an ambient light sensor. It communicates with an STM32 control board (STMicroelectronics, Geneva, Switzerland) via the Modbus protocol through an RS-485 interface to achieve efficient data transmission and processing. After sensor signal processing, the control board transmits data to a serial display interface for real-time visualization.

### 2.2. Experimental Data Collection

The acquired data included infrared thermal images, rectal temperature records, and environmental parameters, together forming a multimodal dataset. To enhance temporal and environmental representativeness, data collection was performed in two stages: the first stage (late spring to early summer 2024) established baseline data, and the second stage (late winter to early spring 2026) supplemented data under broader environmental conditions, especially low-temperature scenarios. Sampling was conducted in a completely randomized order throughout the entire process. Environmental conditions such as piggery ventilation and lighting were maintained constant during the sampling period. Identical experimental protocols were strictly followed in both phases to ensure data homogeneity. Individuals that had been fed within 1 h, transferred between pens within 24 h, or exhibited clinical symptoms were excluded from sampling to eliminate confounding effects of stress and health status. Each sow was measured only once per collection cycle, and the two collection cycles involved completely distinct sow populations.

A total of 968 valid sow samples were obtained, consisting of 2904 high-quality infrared thermal images (968 sows × 3 anatomical regions), along with 968 groups of synchronously measured rectal temperature and multi-dimensional environmental parameters (ambient temperature, humidity, wind speed, and light intensity). The two-stage data collection effectively broadened the coverage of environmental parameters, providing a reliable basis for model training and validation under diverse seasonal and climatic conditions.

(1) Infrared Image Acquisition of Key Regions

The supporting demo software of the Guide IR IPM630 thermal imager was used for infrared image collection. Before shooting, normal communication between the device and the computer was ensured. The emissivity was set to 0.98 to reduce hair interference, and the shooting distance was maintained within 0.5–0.8 m. Preliminary experiments confirmed that this distance achieves a balance between image clarity and anatomical integrity. For each sow, infrared images of the eye, ear, and vulva regions were captured from frontal, overhead, and rear perspectives, with three images collected per individual.

(2) Rectal Temperature Data Acquisition

Veterinary mercury thermometers were used for temperature measurement. All operators received unified standardized training and passed the qualification assessment. A double-reading verification system and regular cross-comparison mechanism were implemented throughout the entire process to effectively eliminate inter-operator reading deviations. Before measurement, the thermometer was cooled to below 35 °C, disinfected with 75% medical alcohol, and calibrated to meet the error tolerance of standard mercury thermometers (accuracy ±0.05 °C). During measurement, the thermometer was inserted into the rectum to a depth of 5–7 cm and held steadily for 3–5 min. Each sow was measured twice with an interval of no less than 2 min, and the average value was recorded as the final result. If the difference between the two measurements exceeded 0.3 °C, an additional measurement was performed to ensure data accuracy.

(3) Environmental Information Collection

During rectal temperature measurement, the environmental data acquisition system synchronously recorded ambient temperature, humidity, wind speed, and light intensity in the pen area. The sensor was positioned 10–20 cm above the sow’s back, enabling simultaneous capture of both the thermal radiation from the body surface and the microclimate of the activity area, thereby minimizing measurement errors. Each environmental parameter was measured in triplicate and averaged, and the corresponding measurement time was recorded simultaneously.

### 2.3. Thermal Image Dataset Preparation and Augmentation

Ear images were annotated with the LabelMe (v5.9.1, GitHub, 2025) polygon tool (JSON format), while the eye and vulva regions were annotated with the LabelImg (v1.8.6, GitHub, 2025) bounding box tool (PASCAL VOC format). To ensure objectivity in model evaluation, the training, validation, and test sets were partitioned at an 8:1:1 ratio at the pig level to avoid cross-contamination. To mimic real-world farming environmental disturbances such as dust, moisture, and motion blur, the training set was augmented via vertical flipping, random rotation, scaling, Gaussian and Poisson noise addition, sharpening, and median blurring, increasing the number of images per anatomical region from 968 to 7744. Augmented images were used exclusively for model training, while both the validation set and test set retained the original images to ensure an unbiased evaluation of generalization ability in real-world scenarios.

### 2.4. Correlation Analysis Between Environmental Parameters and Rectal Temperature

To evaluate the contribution of environmental parameters to sow rectal temperature prediction and to identify effective modeling features while reducing model redundancy, Pearson correlation analysis with two-tailed *t*-tests was performed to examine the linear correlations between ambient temperature, ambient humidity, wind speed, illumination, and rectal temperature (Table 1).

As shown in Table 1, wind speed and ambient temperature are strongly positively correlated with rectal temperature, whereas ambient humidity exhibits a moderately strong negative correlation. These three variables—wind speed, ambient temperature, and ambient humidity—contribute significantly to the prediction of sow body temperature and should be retained as model input features. The correlation coefficient of illumination is merely −0.0222 (*p* = 0.6577), which lacks statistical significance and does not practically contribute to the prediction results. Therefore, illumination can be excluded to reduce model complexity while preserving prediction accuracy. From the perspective of the physiological mechanism of body temperature regulation in pigs, core body temperature is primarily regulated by the balance between body heat production and convective/evaporative heat loss. Light only affects the activity rhythm of pigs and does not directly participate in the body temperature regulation process, so its physiological regulatory effect on rectal temperature is negligible [12,21]. Although light was excluded from the feature set due to its extremely weak correlation with rectal temperature, it may still affect image quality. In this study, all thermal images were collected under standardized lighting conditions and at a fixed shooting distance. Furthermore, infrared thermal imaging relies on thermal radiation emitted by objects themselves rather than reflected visible light, hence the impact of light on temperature measurement or region detection is minimal. Therefore, the exclusion of the light intensity feature has sufficient scientific validity and rationality.

## 3. Fusion Regression Model for Multi-Source Heterogeneous Data

### 3.1. Overall Process

**Figure 1:**
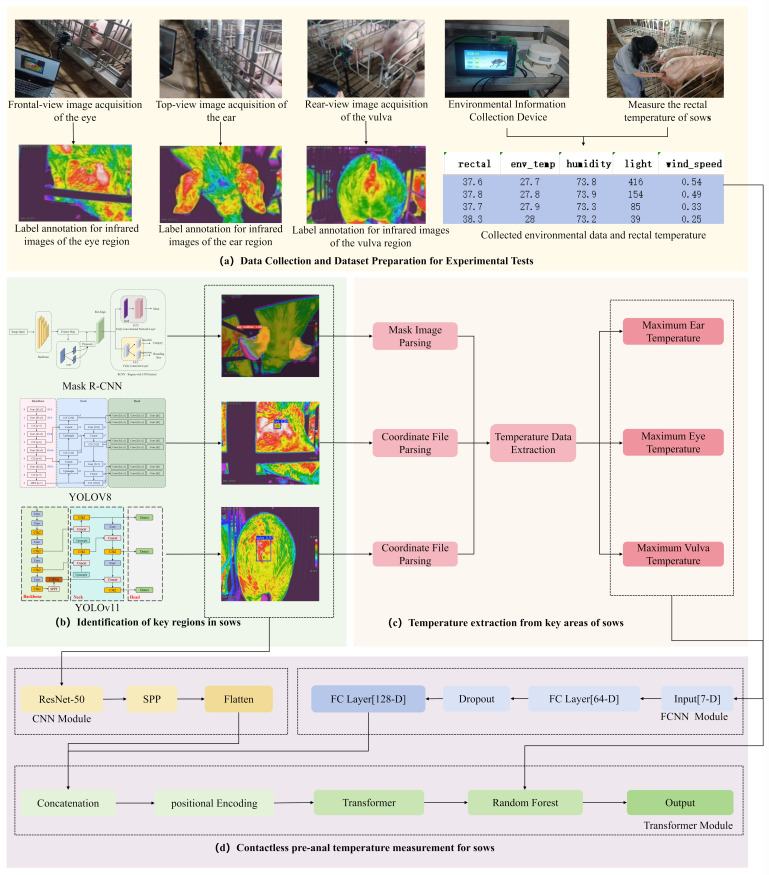
Non-destructive Rectal Temperature Measurement Flowchart for Sows (Arrows guide the order of data processing, color blocks represent different models).

The overall workflow of the proposed multimodal two-stage prediction model (Figure 1) begins with infrared thermal images of three porcine body regions (ear, eye, and vulva) being processed via deep learning-based object recognition. Based on the localization results, the maximum temperature of each region is automatically extracted and regarded as the core numerical feature. Subsequently, a Convolutional Neural Network (CNN) is adopted to extract spatial features from infrared thermal images, while a Fully Connected Neural Network (FCNN) encodes structured variables, including environmental parameters and regional maximum temperatures.

The two-stage prediction architecture is established as follows. In the first stage, the global modeling ability of the Transformer is utilized to generate preliminary rectal temperature predictions. In the second stage, the preliminary outputs are fused with the encoded structured features and then imported into a Random Forest regressor for final prediction refinement. This framework takes full advantage of the complementary merits of deep learning and ensemble learning. The entire model is trained with a five-fold cross-validation strategy to avoid prediction bias. Meanwhile, a multi-random-seed ensemble scheme is applied to stabilize model outputs, thereby achieving end-to-end high-precision prediction of porcine rectal temperature.

The hardware platform for image acquisition and algorithm development consists mainly of a Guide IR IPM630 infrared thermal imager, a custom-built synchronous environmental data acquisition device, and a desktop computer configured with a 12th Gen Intel Core i9-12900K CPU, 32 GB RAM, and an NVIDIA GeForce RTX 3090 (24 GB) graphics card. The software environment is built on Python 3.8 and OpenCV 4.10, with PyTorch 2.0 adopted as the deep learning framework.

### 3.2. Identification of the Thermal Window Region in Infrared Thermal Images of Pigs

#### 3.2.1. Pig Ear Region Segmentation

The pig ear has a distinguishable contour but is easily occluded by breeding fences and affected by variations in head posture. Given the need to preserve the integrity of temperature information, Mask R-CNN was adopted for ear region recognition to achieve precise pixel-level segmentation. Mask R-CNN adopts ResNet-FPN as the backbone network, fusing multi-scale features through the lateral connections of the Feature Pyramid Network (FPN) to accommodate ear size variations under different postures. Instead of the conventional Region of Interest (RoI) Pooling, Mask R-CNN employs RoI Align, which uses bilinear interpolation to accurately compute feature values for candidate regions and eliminates spatial misalignment between the feature maps and the original images. The Fully Convolutional Network (FCN) mask branch works in parallel with the classification and regression branches. Through pixel-wise Softmax classification, precise pixel-level segmentation of the pig ear region is achieved while accurate spatial location information is preserved. This approach effectively satisfies both the morphological integrity and segmentation accuracy required for pig ear extraction.

#### 3.2.2. Pig Eye Target Recognition

To address the detection challenges posed by blurred boundaries between the pig eye region and the facial background, vulnerability to eyelash occlusion, and small target size, the YOLOv8s single-stage object detection model is adopted for recognition. YOLOv8s adopts an enhanced CSP DarkNet53 backbone network integrated with an FPN + Path Aggregation Network (PAN) feature fusion architecture. It utilizes the SPPF (Spatial Pyramid Pooling-Fast) module to mitigate computational complexity and incorporates the VFL (Varifocal Loss) and DFL (Distribution Focal Loss) loss functions to address category imbalance and bounding-box localization deviations, respectively. This method facilitates efficient multi-scale feature fusion and accurate eye region detection.

#### 3.2.3. Pig Vulva Target Recognition

To address the detection challenges posed by blurred boundaries in infrared imaging and tail occlusion in the pig vulva region, the YOLOv11s single-stage object detection model was adopted for recognition. As an optimized successor to YOLOv8s, YOLOv11s replaces the original C2f module with the C3K2 module, enabling efficient feature extraction through smaller convolutional kernels. It integrates the C2PSA spatial attention mechanism to enhance feature capture for occluded targets and complex backgrounds. Moreover, a dynamic label assignment strategy and a lightweight classification head are adopted, which enhance detection accuracy without increasing model complexity. This design effectively meets the detection requirements for the pig vulva region.

### 3.3. Temperature Extraction from Thermal Window Regions

#### 3.3.1. Infrared Image Temperature Matrix Analysis

The infrared images captured by the Guide IR IPM630 thermal imager are stored in the DLT-664 format. This format comprises a file header (the initial 128 bytes containing metadata such as version number, resolution, and acquisition time) and temperature data. Through the reading of the file header, parameters including resolution and data offset can be acquired, facilitating the determination of the starting position of the infrared temperature matrix data (IRData). Subsequently, the data is read line-by-line in floating-point format to generate the temperature matrix. The extraction process is as follows:

(1) File header analysis: Read the first 128 bytes of the file header to obtain parameters such as the width (W) and height (H) of the temperature matrix, as well as the data offset (Offset).
(2) Data localization: The offset determines the starting address of the infrared temperature matrix data (IRData), which is presented as a consecutively stored array of floating-point numbers.
(3) Matrix construction: Read the data row by row in accordance with the resolution to generate an H × W temperature matrix T ∈ R^{H×W}^, where T(i,j) represents the temperature value at pixel (i, j).

#### 3.3.2. Region-to-Matrix Temperature Mapping

Based on the characteristics of different anatomical regions, a differentiated mapping strategy is employed for the image regions and the temperature matrix.

(1) Ear region (segmentation mask-driven)

The Mask R-CNN model is employed for instance segmentation of the ear, producing a binarized mask image M ∈ {0,1}^H×W^, where M(i,j) = 1 indicates that the pixel belongs to the ear region. By traversing the mask image, the coordinates of all pixels with M(i,j) = 1 are extracted, forming the set P_ear_ = (i,j); the corresponding values in the temperature matrix are represented by Equation (1).

(1) $$\left\{T(i,j)|(i,j\right)\;\in {\;P}_{\mathrm{ear}}\}$$

(2) Eyes and vulvar region (test frame driver)

The YOLOv8s (eyes) and YOLOv11s (vulva) object detection models were employed, respectively, to output rectangular box coordinates (x1, y1, x2, y2) (upper left and lower right corners). For the eye region, all pixel coordinates within the rectangular box were extracted; the same procedure was applied to the vulva region, as described in Equations (2) and (3), yielding the coordinate sets P_eye_ and P_vulva_.

(2) $$P_{\mathrm{eye}}=\{\left(i,j)\right|x_{1}\leq j\leq x_{2},\;y_{1}\leq i\leq y_{2}\}$$

(3) $$P_{\mathrm{vulva}}=\{\left(i,j)\right|x_{1}\leq j\leq x_{2},\;y_{1}\leq i\leq y_{2}\}$$

The corresponding sets of temperature values are represented by Equation (4) and Equation (5), respectively.

(4) $$\left\{T(i,j)|(i,j)\in P_{\mathrm{eye}}\right\}$$

(5) $$\left\{T(i,j)|(i,j)\in P_{\mathrm{vulva}}\right\}$$

(3) Core Algorithmic Process

The algorithm uses infrared images as input and outputs temperature statistics for each region (maximum temperature T_max_, minimum temperature T_min_, and average temperature T_mean_). The specific steps are as follows:

  ① File verification: Read the file and verify compliance with the DLT-664 format to ensure data integrity;
  ② Data preprocessing: Parse the file header to obtain resolution and offset values, then read the temperature matrix;
  ③ Model inference: The Mask R-CNN model outputs ear masks, while the YOLO model outputs detection boxes for eyes and vulva;
  ④ Coordinate mapping: Convert masks or detection boxes into pixel coordinate arrays and filter out invalid coordinates;
  ⑤ Temperature extraction: Sample temperature values from the temperature matrix based on the coordinate arrays to generate temperature arrays T for each region;
  ⑥ Statistical calculations: Calculate $T_{\max}\;=\;\max(T),\;T_{\min}\;=\;\min(T)$, $\bar{T}\;=\;\mathrm{mean}(T)$.

### 3.4. Multimodal Two-Stage Pig Body Temperature Prediction Model

#### 3.4.1. Image Feature Extraction Using CNN

Given the distinctive thermal distribution patterns in the thermal window regions of pig infrared images and the need to capture the correlation between local subtle thermal responses and global thermal distribution, the ResNet-50 architecture was selected as the backbone network. This architecture alleviates the gradient vanishing problem in deep network training through residual connections, enabling more efficient extraction of hierarchical features from complex infrared images than traditional convolutional neural networks (CNN). Moreover, the final fully connected layer and global average pooling layer are removed, thereby preserving high-resolution feature maps and preventing the loss of crucial thermal details. The extraction procedure is as follows: (1) ResNet-50 processes the input three-channel infrared images at a resolution of 640 × 512, yielding feature maps of size 20 × 16 × 2048 (H × W × C). (2) To capture thermal features at different scales (e.g., localized hotspots at the ear base or the overall thermal distribution of the ear), a spatial pyramid pooling (SPP) module is incorporated. This module uses 1 × 1, 2 × 2, and 4 × 4 multi-scale pooling kernels to compress features from different body regions into fixed-length 2048-dimensional vectors. (3) Feature vectors from the three body regions are integrated via concatenation to form a unified 6144-dimensional (2048 × 3) image feature vector, comprehensively representing the biological specificity of the pig surface thermal distribution. To avoid losing subtle thermal differences across anatomical regions and physiological information, no further dimensionality reduction was performed on the feature vectors.

#### 3.4.2. FCNN Encoding of Maximum Temperature and Environmental Parameters

The input consists of six-dimensional numerical data with substantially distinct dimensions (e.g., temperature in °C and illumination in lx). Initially, Z-score normalization is applied to eliminate dimensional effects and standardize the data distribution. The encoding network adopts a two-layer fully connected architecture with regularization. Specifically, the first fully connected layer maps the six-dimensional input to 64 dimensions, and the second layer further expands it to 128 dimensions, gradually enhancing the capacity to capture implicit correlations between environmental parameters and body temperature. To mitigate overfitting, Dropout layers (dropout rate = 0.2) and Batch Normalization layers are inserted between the two fully connected layers. Batch Normalization accelerates training convergence by normalizing feature distributions and helps prevent model instability caused by input distribution shifts. The ReLU activation function is employed for its nonlinearity, which effectively captures the complex mapping between environmental parameters and body temperature.

#### 3.4.3. Two-Stage Mixed Prediction Model

Single deep learning regression models are susceptible to high-dimensional image feature noise and environmental perturbations, suffering from large prediction variance and poor generalization ability; whereas traditional machine learning models struggle to extract deep thermal texture features from infrared images. To address this, this study combines the advantages of deep learning in feature modeling and the robustness of ensemble learning against perturbations, and designs a two-stage hybrid architecture consisting of Transformer-based primary prediction and Random Forest-based refined decision-making. This two-level complementary optimization resolves the inherent limitations of single-stage models.

(1) Transformer-Based Cross-Modal Feature Fusion and Primary Prediction

To achieve a unified representation and preliminary regression of heterogeneous features, the process begins by concatenating the 6144-dimensional joint image features from the CNN module with the 128-dimensional environmental encoding features from the FCNN module. This generates a 6272-dimensional cross-modal fusion vector, enabling the initial integration of heterogeneous information. Subsequently, sinusoidal position encoding is applied to incorporate sequential positional information into this fusion vector, converting it into a sequence format compatible with the Transformer architecture input.

The serialized features are then fed into a three-layer stacked Transformer module. In this module, the multi-head self-attention mechanism adaptively assigns weights across different feature dimensions, dynamically modeling the complex global dependencies between image thermal characteristics and environmental parameters. In particular, under conditions of intricate nonlinear coupling between environmental parameters and body surface temperature, this mechanism effectively captures higher-order interaction features, thereby enhancing the representational capacity of the primary predictions.

After processing by the regression head, the Transformer encoder output is passed through a fully connected layer and mapped to a one-dimensional output—the initial rectal temperature prediction (first-stage estimate).

(2) RF-Based Meta-Feature Enhancement and Final Decision

To enhance the stability of initial predictions and fully exploit their informational value, Random Forest (RF) is introduced as a second-stage ensemble learning tool. First, the primary rectal temperature predictions from the first-stage Transformer module are treated as meta-features encapsulating the deep representations learned by the deep learning model. Subsequently, these meta-features are concatenated with the six original standardized structural parameters to construct a 7-dimensional augmented feature vector. Finally, this augmented feature vector is fed into the Random Forest regressor, using the ground-truth rectal temperature as the target for both training and prediction. By constructing an ensemble of decision trees and aggregating their outputs, the Random Forest can effectively capture the complex nonlinear interactions among various environmental parameters. Meanwhile, its Bagging mechanism significantly reduces prediction variance, thereby enhancing the generalization capability and robustness of the final predictions.

#### 3.4.4. Model Training and Ensemble Optimization

Under the PyTorch framework, we constructed a multimodal two-stage hybrid model and adopted stage-wise training, cross-validation, and multi-model ensemble strategies to enhance the rigor of model training, reduce stochasticity, and improve the robustness of predictions. To address the overfitting problem that may be caused by high-dimensional features, this training strategy effectively mitigates it through multiple regularization mechanisms. The detailed training procedure is as follows:

(1) Cross-validation training of the Transformer module

To construct input features without information leakage for the subsequent Random Forest models, the Transformer cross-modal fusion module was trained via five-fold cross-validation. All training data were evenly partitioned into five mutually exclusive subsets. In each round, four subsets were used to train the Transformer model, while the remaining subset was used to generate initial predictions. After five rounds, the predictions from each validation set were aggregated to form an out-of-bag prediction set covering all training samples. This set served as the core feature set for the subsequent Random Forest, effectively preventing overfitting.

(2) Training of the Random Forest module

The out-of-bag predictions generated by the Transformer module were concatenated with the standardized six-dimensional structured parameters to form a seven-dimensional enhanced feature training set. Using actual rectal temperature as the label, a Random Forest regressor was trained. Key hyperparameters (e.g., number of decision trees, maximum depth) were configured to ensure model stability (Table 2).

(3) Multi-model ensemble optimization

To reduce model randomness and prediction volatility, a multi-random-seed ensemble strategy was adopted. During the training phase, multiple independent Transformer models were initialized with different random seeds, and each model underwent the aforementioned five-fold cross-validation process to generate the corresponding out-of-bag predictions. In the testing phase, the test data were fed into each model. The arithmetic mean of the resulting predictions was then used as the ensemble-optimized initial prediction, which was subsequently input into the trained Random Forest for final prediction.

(4) Core training configuration of the Transformer module

During the Transformer module training process, an independent validation set was introduced to dynamically monitor the convergence status and promptly optimize hyperparameters to prevent overfitting. The Huber loss [28] was adopted as the loss function to minimize the interference of individual stress-induced samples during body temperature prediction training. The AdamW optimizer was used with a cosine annealing learning rate schedule, which enabled periodic adaptive adjustment of the learning rate across epochs, balancing the initial exploration phase and the subsequent convergence efficiency. The core hyperparameters were tuned over multiple rounds of fine-tuning to determine the optimal configuration (Table 2).

## 4. Results and Analysis

### 4.1. Comprehensive Performance Analysis of Key Body Region Identification Models

All models were trained on the same hardware platform and within a unified training framework. Specifically, the ear region was detected using an image segmentation model, whereas the eye and vulva regions were identified using object detection models. Given the differences in evaluation metrics between the two tasks, model performance was compared in separate tables. Only the recognition results of the best-performing model for each region are presented in the subsequent figures.

#### 4.1.1. Evaluation System and Experimental Conditions

(1) Evaluation Indicator
  ① Segmentation task (ear region): Segmentation accuracy is evaluated using the Mean Intersection over Union (mIoU), Mean Average Precision (mAP), and Accuracy; computational efficiency is assessed by frame rate (FPS) and floating-point operations (FLOPs); deployment cost is reflected by the model parameter size.
  ② Detection task (eyes, vulvar region): Detection accuracy is measured using precision (Precision), recall (Recall), F1 score, and mean average precision (mAP); computational efficiency is evaluated using FPS and FLOPs, while inference time intuitively reflects real-time performance.
(2) Standardize experimental conditions
  ① Segmentation models (U-Net, DeepLab V3+, Mask R-CNN): Training epochs = 500, Batch size = 8, optimizer: SGD (momentum 0.9, weight decay 1 × 10^−4^, initial learning rate 1 × 10^−3^).
  ② Detection models (Faster R-CNN, YOLOv8s, YOLOv11s): Training epochs = 500, Batch size = 8, optimizer: SGD (initial learning rate 0.01), with early stopping mechanism (patience = 100) to prevent overfitting.

#### 4.1.2. Model Performance Comparison

(1) Ear region

Mask R-CNN achieved the best performance in key accuracy metrics (Table 3). Its mean Intersection over Union (mIoU) and mean Average Precision (mAP) significantly exceeded those of U-Net and DeepLabv3+. It precisely delineated the complete ear contour without background redundancy or edge loss. Although DeepLabv3+ excels in inference speed (FPS) and lightweight design, its segmentation accuracy is relatively low. U-Net attained slightly higher accuracy but incurred excessively high computational costs in terms of Floating Point Operations (FLOPs). Further considering the missed detection performance, Mask R-CNN attained a recall of 0.992, resulting in almost no missed detections of ear regions. U-Net also demonstrated high recall, yet this was accompanied by substantial computational cost. DeepLab V3+ had relatively lower recall, indicating a potential risk of missed detections in small-area or edge regions. Based on comprehensive evaluation of all indicators, Mask R-CNN was determined as the optimal segmentation model for ear regions.

(2) Eye region

The ocular region constitutes a small target in infrared images, necessitating a trade-off between detection accuracy and real-time performance. Among the three detection models compared, YOLOv8s achieved the best overall performance, with both precision and recall surpassing those of YOLOv11s and Faster R-CNN (Table 4). Moreover, it achieved an inference time of only 4.8 ms, offering substantial real-time advantages. YOLOv8s achieved a recall of 0.989, successfully identifying almost all eye targets and effectively reducing body temperature prediction errors caused by missed detections of small targets. YOLOv11s had a slightly lower recall of 0.979 but remained robust. Faster R-CNN only attained a recall of 0.551, exhibiting a significant risk of missed detections. Based on comprehensive evaluation of accuracy, recall and real-time performance, YOLOv8s was selected as the optimal detection model for eye regions.

(3) Vulvar region

The vulvar region is frequently obscured by confounding factors such as fencing and fecal matter, necessitating robust model performance to maintain high localization accuracy in complex scenarios. As presented in Table 5, YOLOv11s outperforms both YOLOv8s and Faster R-CNN in terms of precision, recall, and F1-score. This superiority is attributed to its C2PSA attention mechanism, which enhances feature extraction capabilities for occluded targets. Notably, YOLOv11s achieves a recall of 0.966, surpassing the 0.957 achieved by YOLOv8s, indicating that virtually no critical targets are missed. In contrast, Faster R-CNN exhibits a significantly lower recall of 0.736, suggesting a higher risk of missed detections. Given its balanced performance across precision, recall, and adaptability to complex environments, YOLOv11s is identified as the optimal model for vulvar region detection.

#### 4.1.3. Model Effect Visualization

**Figure 2:**
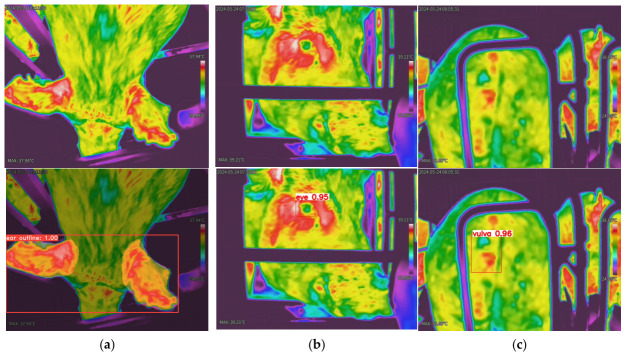
Recognition effects of optimal models for key parts of sows. (**a**) Ear region detection (Original image above, result below); (**b**) Eye region detection; (**c**) Vulva region detection.

The optimal model for each anatomical region (ear: Mask R-CNN; eye: YOLOv8s; vulva: YOLOv11s) was selected for visualization of the recognition performance (Figure 2). The visualizations indicate that all optimal models precisely meet the recognition requirements for their corresponding regions, thus providing a solid foundation for accurate temperature extraction.

Mask R-CNN accurately replicated the natural contours of the ear, with smooth and well-defined boundaries. This effectively eliminated background interference from hair and fences, thereby facilitating precise pixel-level temperature mapping.

YOLOv8s generated detection boxes that were perfectly aligned with the eye region, without any offsets or missed targets. This demonstrated high reliability for small-object detection and met the efficiency requirements of real-time temperature measurement.

YOLOv11s maintained accurate vulva detection even under mild occlusion. The detection boxes fully covered the target area, and the model exhibited robust anti-interference capability, satisfying the complex requirements of livestock farming applications.

### 4.2. Test of the Temperature Extraction Algorithm

To objectively evaluate the accuracy of the temperature extraction algorithm for key body regions, independent infrared thermal images were randomly selected from the test dataset of 30 pigs, comprising 30 ear images, 60 eye images (left and right eyes segmented and analyzed separately), and 30 vulva images. Three researchers manually segmented the target regions using nationally standardized infrared data verification software, and the mean values were used as reference benchmarks to minimize subjective errors in manual annotation. The algorithm’s performance was evaluated using three metrics: Mean Absolute Error (MAE), Pearson Correlation Coefficient (r), and Relative Error (RE).

The algorithm performed best in extracting maximum temperatures (Table 6): the MAE values for maximum temperature in the ear, eye, and vulva regions were 0.2156 °C, 0.0683 °C, and 0.0656 °C, respectively, with correlation coefficients all exceeding 0.96 and average relative errors below 0.6%. In contrast, the extraction errors for minimum and average temperatures were significantly higher, especially for the ear region, where the MAE for minimum temperature reached 1.2655 °C and that for average temperature reached 1.8682 °C.

**Figure 3:**
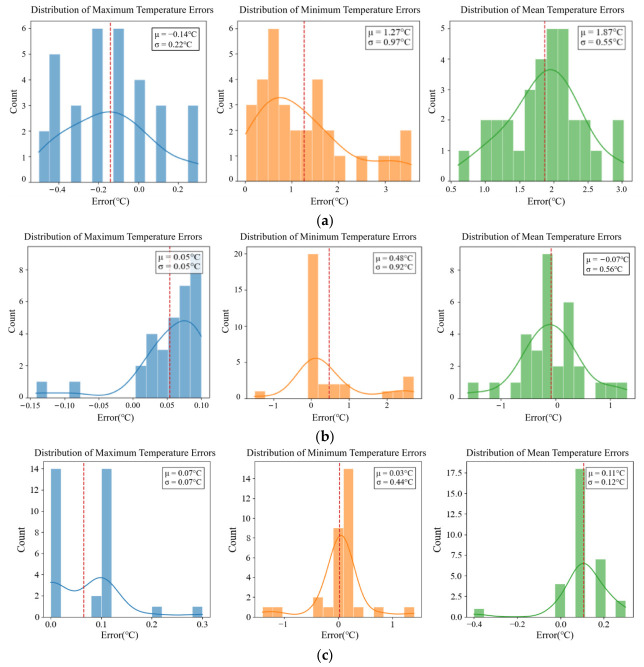
Histogram of temperature error distribution in different areas. (**a**) Histogram of ear error distribution; (**b**) Histogram of eye error distribution; (**c**) Histogram of vulva error distribution.

The error distribution histogram (Figure 3) indicates that 91% of the maximum temperature errors for the ear, 92% for the eye, and 93% for the vulva are concentrated within the ±0.2 °C range. This finding further validates the stability of maximum temperature extraction. The notable errors in minimum and average temperatures mainly originate from three factors. Firstly, in the ear region, the pixel-level mask generated by Mask R-CNN can accurately separate the ear contour from the surrounding hair. In contrast, manual segmentation often incorporates hair areas, which introduces low-temperature points during the minimum temperature measurement, thereby reducing the accuracy of the average temperature. Secondly, the eye region contains only 50–100 pixels, and eyelash interference causes the blurring of infrared image features. This results in a 15–20% boundary deviation between the manual and algorithmic (YOLOv8s) detection boxes, which undermines the consistency of minimum and average temperature extraction. Thirdly, the vulva region is obstructed by tail structures, leading to significant rectangular box positioning errors and an average relative error of 1.3483% in minimum temperature measurement.

**Figure 4:**
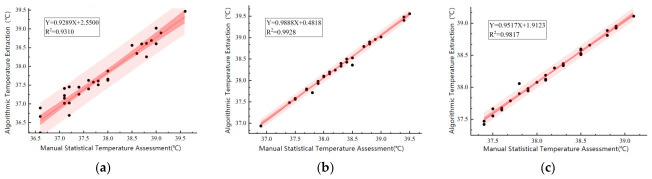
Regression analysis of maximum temperature extracted by algorithm and manually collected. (**a**) Ear; (**b**) Eye; (**c**) Vulva.

The regression analysis (Figure 4) reveals that the regression slopes of the maximum body temperature across various anatomical sites are approximately 1 (ear: 0.9289; eye: 0.9888; vulva: 0.9517), with determination coefficients R^2^ of 0.9310, 0.9928, and 0.9817, respectively, all exceeding 0.92. This confirms the high consistency between the values derived from the algorithm and the manual measurements. From a physiological perspective, the maximum temperature within thermal window regions corresponds to the hotspot areas with the most superficial and dense blood vessels, reflecting the local tissue perfusion and metabolic activities most directly influenced by core body temperature. In contrast, the average temperature is diluted by the cooler peripheral areas within the region, while the minimum temperature is often dominated by artifacts such as hair, moisture, or occlusion, resulting in poor representativeness of core body temperature. Therefore, given its strong anti-interference ability, high correlation with manual measurements, and physiological significance, the maximum temperature is more suitable as a key feature for pig body temperature prediction, which is consistent with mainstream research conclusions in the field of animal thermometry. Meanwhile, the relatively high error in the ear region indicates that anatomical structure and imaging characteristics pose certain challenges to temperature measurement. In practical applications, attention should be paid to the potential impacts of ear hair, optical occlusion, and localization deviation on temperature readings, and prediction reliability can be improved through multiple measurements or by combining information from other anatomical regions.

### 4.3. Performance Analysis of the Multimodal Two-Stage Model

#### 4.3.1. Performance Comparison Between the Multimodal and Benchmark Models

To evaluate the superior performance of the multimodal two-stage prediction model, a systematic comparative experiment was conducted. Four classical single-modal regression models, namely Random Forest Regression (RF), Support Vector Regression (SVR), Gradient Boosting Tree Regression (GBTR), and Multi-Layer Perceptron (MLP), were chosen as comparative benchmarks. All single-modal models were trained with the same six-dimensional numerical features (infrared temperatures of the ear, eye, and vulva, along with ambient temperature, humidity, and wind speed). In contrast, the multimodal two-stage model additionally integrated spatial features from infrared images. All experiments were carried out on the same test set (comprising 150 valid datasets). The coefficient of determination (R^2^) and mean absolute error (MAE) were employed as the primary evaluation metrics (Table 7). The CNN-FCNN-Transformer-RF model outperformed all single-modal benchmark models in terms of both MAE and R^2^. This clearly demonstrates the crucial role of integrating infrared image spatial features with multi-source environmental parameters in improving the accuracy of pig rectal temperature prediction.

#### 4.3.2. Ablation Experiment on the Multimodal Model Architecture

To systematically verify the effectiveness of the Transformer-based primary prediction and Random Forest integration design proposed in this paper, and to explore the optimal model selection strategy for the second stage, this study conducted a multimodal architecture ablation experiment. All comparative models used the same multimodal inputs and training configurations. By comparing the predictive performance of five different architectures, the study quantitatively analyzed the value of incorporating a secondary model and the advantages of heterogeneous integration (Table 8).

The experimental results indicate that all architectures incorporating a two-stage secondary model outperform the single CNN-FCNN-Transformer baseline, confirming the significant value of secondary decision optimization in reducing primary prediction errors and enhancing accuracy. Notably, the CNN-FCNN-Transformer-Transformer model achieved only a 1.58% reduction in MAE compared to the baseline, suggesting that homogeneous model stacking is less effective than heterogeneous integration in balancing bias and variance. In contrast, the CNN-FCNN-Transformer-RF model demonstrated superior performance, with distinct advantages over both homogeneous stacking models and heterogeneous approaches such as MLP and GBR. This fully proves that the Bagging ensemble mechanism of Random Forest can effectively smooth the primary prediction variance of Transformer, making it the optimal secondary ensemble model for Transformer-based primary prediction in the experimental scenario of this paper. In terms of computational cost and real-time performance, the inference time of CNN-FCNN-Transformer-RF was only 13.7 ms per prediction, slightly higher than the baseline but far lower than the 35.2 ms of Transformer stacking. Meanwhile, it maintained low parameter count and Floating Point Operations (FLOPs), striking a balance between prediction accuracy and real-time deployment requirements in large-scale pig farms. In contrast, although MLP and GBR improved accuracy, they incurred higher computational overhead and were difficult to adapt to real-time monitoring in large-scale piggeries. Therefore, CNN-FCNN-Transformer-RF achieved the optimal balance between accuracy and real-time performance in this experimental scenario and is the most suitable secondary ensemble model.

#### 4.3.3. Comparison of Combined Modeling Results for Thermal Window Regions

Based on the test results of the temperature extraction algorithm described in Section 4.2, among the three anatomical regions (ear, eye, and vulva), the ear exhibited the highest temperature extraction error and the lowest coefficient of determination (R^2^) between the extracted and manually recorded values. As a result, its thermal information exhibits lower stability and reliability compared with that of the eye and vulva. To further reveal the physiological mechanism underlying this phenomenon and determine the optimal feature combination, 7 groups of controlled experiments covering single-region, dual-region, and triple-region combinations were systematically designed while keeping other parameters constant. From the perspectives of anatomy and hemodynamics, the fusion of the peri-ocular area and vulva possesses inherent complementary advantages: the peri-ocular area, as a direct surface mapping of core body temperature, is hairless with dense superficial blood vessels and extremely low thermal inertia, serving as a “sensitive indicator” for capturing transient changes in body temperature. In contrast, the vulva is protected by the natural barriers of subcutaneous fat and skin folds, exhibiting extremely strong anti-interference ability against external airflow and humidity, thus constituting a “stable anchor” for core body temperature. The system was modeled precisely based on this physiological complementary characteristic of dynamic capture and steady-state reference. As shown in Table 9, the dual-region scheme that excludes the ear and retains only “peri-ocular area + vulva” outperformed all other combinations in all core performance indicators. This confirms that the strategy of fusing high-sensitivity regions and high-stability regions can effectively balance the response speed and anti-interference ability of temperature measurement.

## 5. Discussion

This study investigated a modeling approach for pig body temperature prediction based on infrared thermal imaging and deep learning. The key findings are as follows:

(1) A differentiated framework for precise perception and automated feature extraction was developed for complex pig-farming scenarios. This framework addresses the limitations of traditional manual box-selection methods, which suffer from low efficiency and poor consistency. By employing customized instance segmentation and object detection models tailored to the unique anatomical and imaging characteristics of the ear, eye, and vulva, the framework achieves high-precision automatic localization and temperature extraction. The extracted results show strong correlations with manual measurements (ear: r = 0.9650; eye: r = 0.9960; vulva: r = 0.9910), significantly enhancing the reliability and automation of feature acquisition and providing a high-quality data foundation for subsequent modeling.
(2) Correlation and significance testing of environmental parameters revealed that wind speed, ambient temperature, and humidity were significantly correlated with rectal temperature, whereas illumination was excluded due to its minimal contribution. This approach reduces model complexity while maintaining prediction accuracy.
(3) This paper presents a multimodal two-stage prediction model that overcomes the performance limitations of traditional single-modal or simple serial models, representing the core algorithmic contribution of this research. The hybrid architecture outperforms pure Transformer models, MLP and GBR models, and homogeneous stacked Transformer architectures, offering a novel modeling approach for complex livestock prediction tasks.
(4) Comparative experiments on thermal window region combinations for pig body temperature prediction revealed that the “eye + vulva” combination achieved superior predictive performance (MAE = 0.1796 °C). This finding offers a reliable theoretical basis for the practical application of non-invasive temperature measurement technologies.

## 6. Conclusions

This study addresses the core bottleneck of non-invasive porcine body temperature monitoring under complex farming conditions and develops a multimodal two-stage deep learning framework that integrates infrared thermal imaging with multi-source environmental data. The key finding is that the proposed CNN-FCNN-Transformer-RF model, using the “eye + vulva” dual-region combination, achieves a mean absolute error of 0.1796 °C and a coefficient of determination of 0.8212, outperforming single-modal and single-region benchmarks by a substantial margin. The core practical contribution of this work is to provide a rapid, accurate, and stress-free solution for sow body temperature monitoring, which supports the development of automated and animal-friendly intelligent health management systems in livestock farming.

However, several challenges remain for practical deployment. Animal movement and posture changes may cause motion blur or thermal window region shifts; occlusion by fences or other pigs may temporarily block key regions; camera positions and angles need to be optimized to continuously capture the eyes, ears, and vulva. Although light has minimal impact on thermal imaging, extreme light variations (such as direct sunlight and dark environments) may affect image contrast and detection reliability. Furthermore, the limited representativeness of the current dataset—derived from a single breed and a specific farm—may constrain the model’s generalizability to diverse production systems. The hardware costs of high-resolution thermal cameras and computing devices, as well as long-term model maintenance (e.g., retraining for new farms or new breeds), also need to be considered. Future work will focus on: (i) developing real-time tracking and motion compensation algorithms to improve temperature measurement stability in dynamic scenarios; (ii) designing automatic quality assessment modules to reject occluded or blurry frames; (iii) optimizing camera layouts and exploring multi-view fusion to improve coverage of key regions; (iv) developing lightweight models and exploring edge computing deployment to reduce hardware costs and enable large-scale real-time monitoring; (v) establishing continuous learning pipelines to enable adaptive model updates over time and enhance generalization ability across farms and breeds; (vi) exploring the integration of body temperature prediction with other health parameters (e.g., activity level and water intake) to achieve more comprehensive intelligent health management.

## Figures and Tables

**Table 1:** Correlations between environmental parameters and rectal temperature in sows.

Environmental Parameter	Pearson Correlation Coefficient r	Correlation Direction	Significance Level	Correlation Strength
Wind speed	0.5978	Positive	*p* < 0.001	Strong
Ambient temperature	0.4008	Positive	*p* < 0.001	Strong
Ambient humidity	−0.2327	Negative	*p* < 0.01	Moderately strong
Illumination	−0.0222	Negative	*p* = 0.6577	No correlation

**Table 2:** Hyperparameter table of the multimodal sow body temperature prediction model.

Parameter	Specification
Loss function	Huber Loss
Optimizer	AdamW
Initial learning rate	1 × 10^−4^
Epoch	200
Batch size	8
Cross-validation folds	5
Transformer random seeds	42, 43, 44
Number of decision trees (RF)	100
Maximum depth (RF)	8
Minimum samples per leaf (RF)	4
Minimum samples per split (RF)	2

**Table 3:** Performance Comparison of Sow Ear Region Segmentation Models.

Model Name	mIoU	mAP	Accuracy	Recall	FPS	FLOPs/G	Parameters/M
U-Net	0.973	0.986	0.993	0.981	32.653	564.926	94.95
DeepLab V3+	0.959	0.979	0.989	0.962	104.411	65.857	22.18
Mask R-CNN	0.996	0.988	0.985	0.992	2.025	41.472	63.73

**Table 4:** Performance Comparison of Sow Eye Region Detection Models.

Model Name	Precision	Recall	F1-Score	mAP	FLOPs/G	Inference Time/ms
Faster R-CNN	0.44	0.551	0.488	0.41	350.25	34.13
YOLOv8s	0.997	0.989	0.993	0.933	78.7	4.8
YOLOv11s	0.991	0.979	0.985	0.903	74.9	5.4

**Table 5:** Performance Comparison of Sow Vulva Region Detection Models.

Model Name	Precision	Recall	F1-Score	mAP	FLOPs/G	Inference Time/ms
Faster R-CNN	0.689	0.736	0.710	0.689	350.25	34.11
YOLOv8s	0.978	0.957	0.968	0.942	78.7	5.3
YOLOv11s	0.991	0.966	0.978	0.948	74.9	6.0

**Table 6:** Correlation coefficient and error statistics table.

Body Part	Temperature Type	MAE/°C	Correlation Coefficient	Min. Relative Error/%	Max. Relative Error/%	Avg. Relative Error/%
Ear	Maximum	0.2156	0.9650	0.0500	1.4059	0.5734
	Minimum	1.2655	0.8710	0.0087	11.4935	3.6033
	Average	1.8682	0.8170	0.1169	7.3563	3.7342
Eye	Maximum	0.0683	0.9960	0.0084	0.3675	0.1788
	Minimum	0.5688	0.7540	0.0304	8.5247	1.7035
	Average	0.4250	0.8460	0.0155	4.4030	1.1994
Vulva	Maximum	0.0656	0.9910	0.0202	1.0632	0.1854
	Minimum	0.2312	0.9780	0.0117	9.9907	1.3483
	Average	0.1312	0.9850	0.0069	4.1743	0.7274

**Table 7:** Performance Comparison of Different Sow Body Temperature Measurement Models.

Model Category	MAE/°C	R^2^
RF	0.3110	0.5127
SVR	0.4013	0.1884
GBTR	0.3675	0.3192
MLP	0.4055	0.5988
CNN-FCNN-Transformer-RF	0.1885	0.8134

**Table 8:** Ablation Study Results of Multimodal Model Architectures.

Model Architecture	MAE/°C	R^2^	Inference Time/ms	FLOPs/G
CNN-FCNN-Transformer (base)	0.2342	0.7285	12.5	2.8
CNN-FCNN-Transformer-Transformer	0.2305	0.7371	35.2	6.5
CNN-FCNN-Transformer-MLP	0.2251	0.7498	18.3	3.2
CNN-FCNN-Transformer-GBR	0.2183	0.7845	22.1	3.6
CNN-FCNN-Transformer-RF	0.1885	0.8134	13.7	2.9

**Table 9:** Performance Comparison of Different Site Combination Modeling.

Input Feature Site Combination	MAE/°C	R^2^
Ear	0.2424	0.6086
Eye	0.2117	0.7103
Vulva	0.2088	0.7192
Ear + Eye	0.1996	0.7465
Ear + Vulva	0.1913	0.7598
Eye + Vulva	0.1796	0.8212
Ear + Eye + Vulva	0.1885	0.8134

## Data Availability

The original contributions presented in this study are included in the article. Further inquiries can be directed to the corresponding author.
